# Latest developments in MUC1 immunotherapy

**DOI:** 10.1042/BST20170400

**Published:** 2018-05-21

**Authors:** Joyce Taylor-Papadimitriou, Joy M. Burchell, Rosalind Graham, Richard Beatson

**Affiliations:** Breast Cancer Biology Lab, School of Cancer and Pharmaceutical Sciences, King's College London, London, U.K.

**Keywords:** cancer, immunotherapy, MUC1

## Abstract

Currently, there is renewed interest in attempting to recruit the host immune system to eliminate cancers, and within this renewed activity, MUC1 continues to arouse interest. MUC1 has been considered a possible therapeutic target for the past 30 years as it is up-regulated, aberrantly glycosylated and its polarization is lost in many adenocarcinomas. Moreover, MUC1 is expressed by some haematopoietic cancers, including acute myeloid leukaemia and myeloma. Although multiple clinical trials have been initiated and immune responses have been documented, effective clinical benefit worthy of approval for general application has not as yet been achieved. However, this does not appear to have quelled the interest in MUC1 as a therapeutic target, as shown by the increase in the number of MUC1-based clinical trials initiated in 2017 ( Figure 1). As with all translational studies, incorporating new relevant research findings into therapeutic strategy is difficult. Decisions are made to commit to a specific strategy based on the information and data available when the trial is initiated. However, the time required for preclinical studies and early trials can render the founding concept not always appropriate for proceeding to a larger definitive trial. Here, we summarize the attempts made, to date, to bring MUC1 into the world of cancer immunotherapy and discuss how research findings regarding MUC1 structure and function together with expanded knowledge of its interactions with the tumour environment and immune effector cells could lead to improved therapeutic approaches.

**Figure 1.**
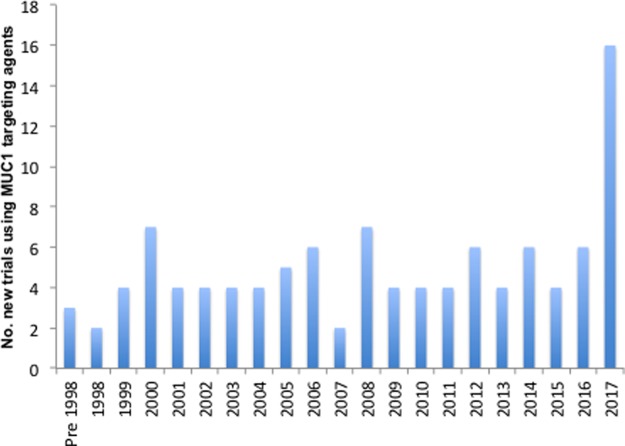
Number of MUC1-targeted trials initiated each year.

## Background

**Figure 2.**
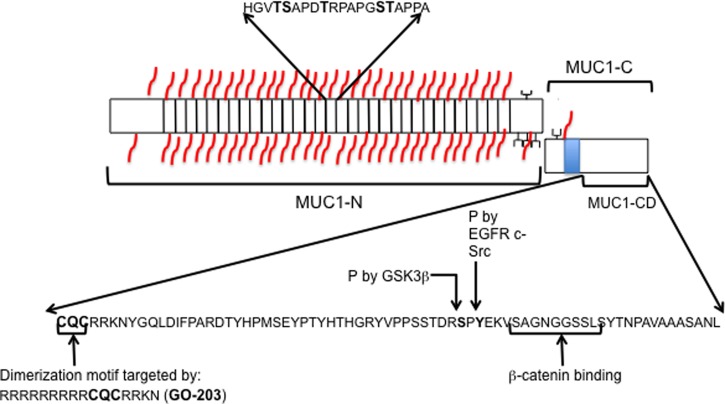
The structure of the MUC1 mucin. During translation, MUC1 is cleaved into two domains, MUC1-N and MUC1-C. MUC1-N consists predominantly of the TR domain and the sequence of a single repeat is illustrated. The TR domain is glycosylated with O-linked glycans (in red) and each repeat has five potential sites shown in bold. There are also sites for O-linked glycosylation in the degenerate TRs located to the N- and C-termini of the repeats. There are five potential sites for N-linked glycosylation close to the membrane (in black). MUC1-C consists of 58 amino acids of the external domain, the transmembrane domain (in blue, 28 aa) and the cytoplasmic domain (MUC1-CD, 72 aa). Within the CD1 domain, the CQC trimer is responsible for homodimerization. There are many phosphorylation sites within the CD domain and two of these are indicated. The CQC containing peptide (GO-232) targets the homodimerization domain.

MUC1 was the first membrane mucin to be identified and the first mucin to be characterized structurally [1–4] (see Figures 1 and 2). The protein is cleaved during translation [5,6], and the tandem repeat (TR) containing external domain (25–100 TRs) is bound to the membrane indirectly by non-covalently interacting with the MUC1-C domain that consists of a short extracellular domain (EC) and the transmembrane and cytoplasmic domains (see Figure 2). MUC1-C is known to be involved in signal transduction [7,8].

**Figure 3.**
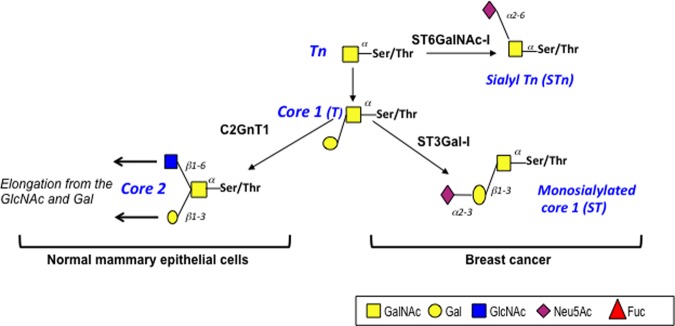
Simplified pathways of mucin-type O-linked glycosylation in normal and malignant breast epithelial cells. The Tn glycan can be carried on MUC1 expressed by a high proportion of breast cancers. The STn glycan is found in ∼25% of breast cancers, while ST is more commonly expressed. The unsialylated core 1 (T) is also widely found on MUC1 expressed by breast cancers. In contrast, the glycans found on MUC1 expressed by normal mammary epithelial cells are core 2 based.

The MUC1 glycoprotein was first identified as the antigen found in extracts of the human milk fat globule membranes, or in extracts of cancer cells, which induced a strong humoral response in mice, leading to the production of many monoclonal antibodies reactive with the TR domain [9–12]. These antibodies were used to clone the MUC1 gene and differences in the reactivity of some antibodies with normal epithelia and carcinomas were observed [13,14]. MUC1 is extensively glycosylated and the differences in antibody reactivity were found to be due to changes in the O-glycosylation of the serines and threonines in the TR domains of the cancer mucin where the glycans added were truncated and more heavily sialylated than in the normal epithelia [15,16] (see Figure 3).

Many of the MUC1-reactive antibodies reacted with peptide sequences in the TR domain, and the concept arose that in the cancer mucin, with shorter glycan chains, some of these peptide sequences were more exposed, thus explaining the tumour specificity of antibodies such as SM3 and DF3-P [17,18]. This led to the belief that peptide antigens based on the MUC1 TR sequences would induce tumour-specific adaptive immune responses. Many small Phase I/Phase II clinical trials using MUC1-based peptide immunogens were initiated, but these have not graduated to larger Phase III trials [19]. This may reflect the paucity of MHC class I epitopes within the TR of MUC1. Nevertheless, four MUC1-based Phase III trials were performed, one of which used an MUC1 peptide as immunogen (L-BLP25) [20]. These trials were completed several years ago and the therapies investigated, outlined in Table 1, are not under further evaluation (see Outcomes and Comments in Table 1).

**Table 1:** Phase III MUC1-based clinical trials

Vaccine	Cancer/number of patients	Treatment	Outcome	Comment	Investigators/year
**Peptide vaccine:** L-BLP25 (tecemotide) Lyophilized 25mer MUC1 TR lipopeptide + lipid adjuvant	*N *= 1513 Stage IIIb NSCLC patients	Administered post chemoradiotherapy versus placebo. Cyclophosphamide administered once before first tecemotide administration, or saline prior to placebo	No overall survival (OS) difference or time to relapse within whole cohort. OS significantly increased in subgroup treated after simultaneous chemo- and radiotherapy with L-BLP25 (*n *=* *538) versus placebo (*n *=* *238): 30.6 versus 20.6 months (*P *=* *0.016)	Claimed that MUC1-specific cytotoxic T cells were induced; however, Class I epitopes for CD8 stimulation are found outside TR. Control group with MUC1 liposomes not included therefore unclear of contribution of lipid stimulation of Toll receptor versus MUC1 effects.	[20]
**Glycan vaccine:** Sialyl Tn-KLH	*N *=* *1028 Breast cancer patients	Sialyl Tn-KLH versus KLH alone. Cyclophosphmide included in all treatments to down-regulate Tregs	No significant difference in OS in patients receiving sialyl Tn-KLH versus KLH alone	Only 20–25% of breast tumours express sialyl Tn and expression of STn on patients tumours was not assessed. Still unclear whether vaccine could be effective in patients with STn-positive cancers	[62]
**Viral vaccine:** PANVAC-VF viral vector expressing CEA and MUC1 plus B7.1, intracellular adhesion molecule-1 and leukocyte function-associated antigen-3	*N *=* *255 Advanced pancreatic cancer patients	PANVAC-VF versus palliative chemotherapy	No significant difference in OS of patients receiving PANVAC-VF versus palliative chemotherapy or best supportive care	Plans to file Biologics License Application (BLA) with FDA abandoned	Therion Unpublished www.medicalnewstoday.com/releases/46137 June 2006
**Antibody vaccine:** murine MAb HMFG1 carrying Yttrium-90	*N *=* *447 ovarian cancer patients in remission after surgery and chemotherapy	Single dose ^90^Y HMFG1 versus placebo	No OS or time to relapse seen in antibody-treated group compared with placebo	HMFG1 does not show good specificity for cancer-associated mucin. Use of a murine Ab is not appropriate. HMFG1 was subsequently humanized and retried in phase II (AS1402 and BTH1704) before being discontinued	[63]

Abbreviations: TR, tandem repeat; KLH, keyhole limpet haemocyanin; MAb, monoclonal antibody; OS, overall survival; STn, sialyl Tn; CEA, carcinoembryonic antigen.

## Phase I/II trials reported in the literature

### The viral vaccine TG4010

*The viral vaccine TG4010* developed by Transgene has shown promising results and is moving to further trials. TG4010 is a modified Vaccinia Ankara strain expressing a full-length MUC1 (containing five TRs) and IL-2. The Phase IIb trial in non-small cell lung cancer (NSCLC) patients was completed, where the viral vaccine was administered in combination with chemotherapy [21]. Response to the viral vaccine was predicted by levels of natural killer (NK) cells and TG4010 plus chemotherapy improved progression-free survival compared with chemotherapy and placebo. Further studies on the patients and tumours from this trial indicate that TG4104 induces a broadening of the T-cell response to other tumour-associated antigens and the diversity of the T-cell response relates to response to therapy [22].

With FDA approval, a Phase II clinical trial has been initiated for the first-line treatment of NSCLC patients, exploring the effect of combining *TG4010* with chemotherapy and the Bristol-Myers Squibb's immune checkpoint inhibitor *nivolumab* [23]. This study is now recruiting participants.

**Comment**: After injection of the virus, full-length MUC1 glycoprotein is expressed; therefore, Class I epitopes outside of the TR domain can be presented. Moreover, the epitope spreading to other TAA is a positive parameter. Combining the vaccine with the checkpoint inhibitor could also enhance efficacy.

### Several Phase I/II trials injecting autologous dendritic cell

Several Phase I/II trials injecting *autologous dendritic cell (AuDC)* loaded with MUC1 as a peptide, mRNA or fused to tumour cells have been reported [24–31]. The time taken for the preclinical data using dendritic cells (DCs) fused to tumour cells to be translated to the clinic and show efficacy [28,29] illustrates the length of time required for incorporating research into the clinic.

The trials using AuDCs loaded with oxidized mannan linked to a 5TRMUC1 peptide also show promise. Unusually, this trial in breast cancer patients has a 15-year follow-up [32] when the recurrence rate in patients receiving placebo was 60% (nine of 15) compared with 12.5% (two of 16) for those receiving immunotherapy.

**Comment for the oxidized mannan trial:** This immunogen is now called CVac by the company Prima Biomed, which is no longer recruiting patients but is seeking commercial partners for the product.

### Adoptive immunotherapy with Gemcitabine

The investigators of the present study specify that it was not a trial but a medical treatment approved as advanced health care by the Japanese Ministry of Health, Labor and Welfare, and patients were self-funded. Forty-two late-stage pancreatic patients were given injections of autologous DCs pulsed with full-length MUC1 mRNA and autologous CTLs generated *in vitro.* Average mean survival time (MST) for all patients was 13.9 months, and the 1-year survival rate was 51.1%. For patients (*n* = 30) who received more than 1 × 10^7^ MUC1-DCs per injection and 3 × 10^8^ MUC1-CTLs per injection, MST was 16.5 months versus MST of 5.7 months in the other 12 patients (*P *= 0.00020) [33].

**Comment:** CTLs were generated using one cell line — YPK-1 (HLA-A2402) — assuming the generation of Class I non-restricted MUC1-reactive CTLs. Such CTLs have been reported [34], but they have proved to be difficult to characterize. Nevertheless, the study documents effective immune responses to MUC1.

## Preclinical studies and combination therapies

Preclinical studies continue to be initiated using various vaccine formats of MUC1 based on the glycosylated or unglycosylated TR sequence. Investigators in the Kunz laboratory have chemically synthesized a wide range of glycopeptides based on the TR of MUC1 and have evaluated various formulations of these (containing factors to recruit innate immunity and/or CD4 help) for antibody induction in WT mice [35,36]. Some of these new antibodies show specificity for the tumour MUC1 glycoforms [37] and humoral and cellular immune responses to one vaccine formulation (1TR carrying Tn and STn bound to tetanus toxin) have been detected in MUC1 transgenic mice where human MUC1 is expressed from the MUC1 promoter [38].

A study using MUC1 transgenic mice for evaluation of the effect of an anti-PD-L1 antibody on the growth of an aggressive MUC1 expressing murine ovarian cancer cell line (2F8) is illuminating [39]. Tumour-bearing MUC1 transgenic mice treated with anti-PD-L1 antibody (21-day post tumour injection) showed increased survival and T-cell infiltration within the tumour, but induction of MUC1-specific antibodies was low.

Combination immunotherapy (using checkpoint inhibitors together with MUC1 strategies) is now being initiated as well as testing MUC1 as an immunogen together with other antigens (https://clinicaltrials.gov/), but data are not yet available. If several antigens are presented, interpretation regarding the contribution of MUC1 to effects on tumour growth, which may be documented, will be difficult.

## Applying research findings to immunotherapeutic strategies

Apart from the developments on immune checkpoint inhibitors, investigations into the interactions of cancers with the immune system and with their local environment are leading to other important findings. Information relating to the importance of the change in glycosylation of MUC1 and the interaction of specific cancer MUC1 glycoforms with cells in the microenvironment are also suggesting novel approaches. Moreover, a role for the MUC1-C component is becoming of central importance.

### Importance of aberrant glycosylation of MUC1 in immunotherapy

A role for glycopeptide epitopes in inducing not only humoral but also cellular adaptive immune responses has been recognized for many years [40], but it is only relatively recently that the focus on glycosylation changes has been seriously pursued in the search for effective MUC1-related immunotherapy.

#### Antibodies to glycopeptide epitopes in the TR domain

##### PankoMab-GEX

Many MUC1-specific antibodies react with epitopes within the PDTRP sequence and reactivity is affected by glycosylation [11,17,18,41]. The PankoMab antibody reacts with a conformational epitope where the threonine in PDTRP carries the Tn or T glycan and selectively reacts with the cancer mucin [42]. PankoMab-GEX has been humanized and glyco-optimized for improved ADCC and ADCP activity and enhanced NK cell killing. In a Phase I trial [43] (NCT01222624), 74 patients with advanced MUC1-positive carcinomas received PankoMab-GEX intravenously in a three-plus-three dose-escalation design until disease progression. PankoMab-GEX showed promising anti-tumour activity in advanced disease and a Phase IIb study is now ongoing evaluating the efficacy of PankoMab-GEX as maintenance therapy in advanced ovarian cancer.

#### Retargeting of human T cells to tumour-associated MUC1 glycoforms

Chimeric antigen receptors (CARs) targeting T cells to MUC1 were first developed and characterized by the Maher group [44] using the SM3 antibody, which is highly selective for cancer MUC1 glycoforms [17], and the HMFG2 antibody, which also shows some selectivity. Several CARs were engineered and the HMFG2 CAR containing a fused CD28 plus OX40 plus CD3 endodomain (HOX HMFG2) together with the IgD linker, to combat glycosylation-independent steric hindrance, was shown to inhibit breast cancer growth in a mouse model.

More recently, a CAR fusion protein with a humanized version of the 5E5 antibody has been developed [45] based on the evidence that 5E5 shows impressive specificity in reacting selectively with the Cancer MUC1Tn glycoform. 5E5 was raised against a 60mer TR peptide carrying five GalNAc residues (Tn) per TR and reacts preferentially with the GSTA sequence carrying Tn and to a lesser extent STn [46,47]. Unlike PankaMab, which recognizes the PDTR epitope carrying either Tn or T, 5E5 does not recognize the GSTA epitope carrying T.

Studies with these CAR constructs have not yet advanced to the clinic: MUC1 targeting CAR cells have been held in the preclinical setting for many years while technical and biological challenges are overcome — not least the expression of MUC1 on T cells themselves [48]. The specificity of the 5E5 CAR, which does not bind the glycoform of MUC1 on T cells [49], could circumvent problems that might be encountered with other MUC1 CARs, which do bind to MUC1 on T cells.

There are 10 active Phase I/II CAR trials targeting MUC1 in multiple solid and non-solid malignancies currently recruiting: nine using α/β T cells and one using NK cells, some of which are trialling combination therapy with checkpoint blockade (https://clinicaltrials.gov/). Identification of the antibodies, from which the CARs were derived, is not always easy and the trials are in regions where the stringency of requirements for cell therapy is not as high as in Europe or the USA. Nevertheless, it will be interesting to see how these trials develop.

#### CD4 T-cell responses to MUC1 glycopeptides

When presented by DCs in MUC1 transgenic mice, MUC- reactive CD4 T-cell responses have been documented [28,50], along with the induction of humoral immunity [28,50]*.* Moreover, several investigators have demonstrated that MUC1 vaccines carrying the Tn and/or STn glycan are not subject to self-tolerance in MUC1 transgenic mice [38,46,51].

These observations are now being taken further along the road to clinical evaluation and a small Phase I clinical trial has been reported where a DC vaccine loaded with 100mer TR MUC1 peptide carrying Tn was administered with KLH to non-metastatic, castrate-resistant prostate cancer patients [52]. This trial was preceded by a study in Rhesus Macaques where the corresponding Tn-rmMUC1 in autologous DCs was administered with an adjuvant and induction of antibodies and T-cell activation monitored. The rmMUC1 study demonstrated T-cell activation but MUC1-reactive antibodies were not induced. The same profile of activation of the adaptive immune response was seen in the prostate cancer patients: predominantly, CD4 T-cell responses were seen with CD8+ responses being variable, while no MUC1-reactive antibodies were induced.

Eleven of 16 patients treated showed an increase in PSA doubling time (10.5 months) compared with the pre-vaccination value (4.6 months) .

### MUC1 effects on the tumour environment and immune effector cells

It is becoming apparent that MUC1 expressed by cancer cells can affect the phenotype and function of immune cells in the tumour microenvironment inhibiting their function.

#### Effects of a cancer glycoform of MUC1 on myeloid-derived cells

• *The dominant glycoform of MUC1 expressed by most carcinomas is MUC1-ST* (see Figure 3), which is expressed on the cell surface and can be secreted. Beatson et al. have shown that MUC1-ST interacts with the sialic acid-binding protein Siglec-9 expressed on monocytes and macrophages: this interaction induces the development of tumour-associated macrophages showing increased expression of the PD-L1 checkpoint ligand as well as other proteins associated with tumour growth and metastases [53]. Many proteins of the Siglec family of sialic acid-binding proteins are involved in immune suppression of innate and adaptive immune effector cells and tumours exploit these ‘Glycoimmune checkpoints’. The Palleon Pharmaceutical Company is developing antibodies to block the glycan–Siglec interactions, including that of Siglec 9 with MUC1-ST (Palleonpharma.com).

• *Induction of myeloid-derived suppressor cells* (*MDSCs*) by MUC1 in acute myeloblastic/myelogenous leukaemia (AML) cells has also been reported [54]. MDSCs, which derive from immature monocytes and suppress effector T cells, were induced by extracellular vesicles from AML cells. MUC1 is a critical component of these vesicles and up-regulates c-MYC expression by down-regulating the expression of MiR34 that targets c-MYC. By silencing expression of MUC1, c-MYC target genes are suppressed in the recipient myeloid cells and levels of MDSCs are significantly reduced. Although not specifically shown to be the domain of MUC1 responsible for the generation of MDSCs, the MUC1-C cytoplasmic component is a candidate. Moreover, this component of MUC1 has been shown to contribute to immune evasion in other ways ([55] and see below).

### Targeting the transmembrane component of MUC1 (MUC1-C)

Although much of the work relating to MUC1 as a therapeutic target in cancer has focused on the EC domain, interest in the smaller membrane spanning component (MUC1-C) has increased.

Preclinical studies showed that MUC1-C interacts with EGFR and other membrane receptors and that the cytoplasmic domain is transported to the nucleus where it activates multiple signalling pathways [7]. Consequently, a peptide inhibitor (GO-203) has been developed and directed to the CQC sequence in the cytoplasmic domain of MUC1-C, which is required for dimerization and signalling activity (Figure 2). Using CRISPR gene editing and/or the GO-203 inhibitor to silence MUC1 in multiple myeloma cells, MUC1-C was shown to activate MYC expression by binding to the promoter in a β-catenin/transcription factor 4-mediated mechanism [56]. Activation of MYC leads to increased expression of MYC targets including BMI and other components of the Polycomb 1 complex [57].

Clearly, these data indicate that the molecular mechanisms activating expression of MYC are different in cells derived from different tumours (see MUC1, MYC and MDSCs above). This also applies to the mechanisms reported to underlie the induction of the PD-L1 ligand by MUC1 in different tumour types. In NSCLC cell lines, MUC1-C is associated with NF-κBp65 at the promoter of *PD-L1* thus directly affecting transcription and is also associated with immune evasion and a suppressive immune microenvironment [58,59]. On the other hand, in AML cells, MUC1 is reported to inhibit the expression of several microRNAs targeting PD-L1 by inhibiting Dicer [60]. Nevertheless, effects of MUC1-C on PD-L1 induction and immune invasion are widely seen in other cancers, including triple-negative breast cancer [61], and the MUC1-C inhibitor GO-203 has now completed a Phase I trial in patients with advanced solid tumours: patients are now being recruited for a Phase II trial of GO-203 in combination with decitabine for patients with AML (NCT02204085).

## Summary

**Figure 4.**
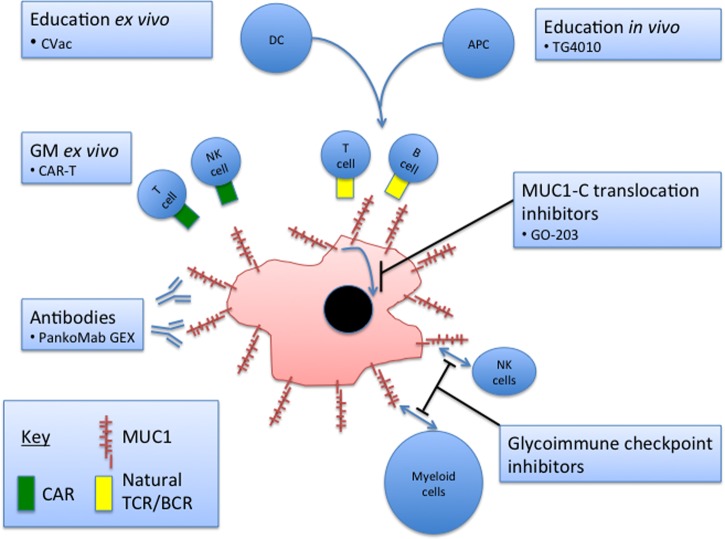
Current and potential clinical approaches targeting MUC1 in cancer. Current strategies fall into six broad categories (as shown in the boxes) ranging from preclinical to Phase III. The bullet point within each box gives an example of that category of immunotherapy. To date, no therapeutic targeting MUC1 has been approved for general use. GM, genetically modified; DC, dendritic cell; APC, antigen-presenting cell; NK, natural killer.

Efforts continue to investigate strategies for exploiting the overexpression and aberrant glycosylation of MUC1 in multiple cancer types and possible approaches to MUC1-based immunotherapy are summarized in Figure 4. Some recently initiated clinical trials are taking on board the novel findings emerging from work on the structure and function of MUC1 and its interaction with the tumour environment and immune effector cells. These data indicate that both the MUC1-N (highly glycosylated) and MUC1-C components contribute to immune evasion by cancer cells, and this could be an important consideration in the strategic development of MUC1-based immunotherapy. The findings emphasize that although testing the *in vitro* findings in clinical trials is important, it is also important to continue preclinical studies aimed at further defining the function of this glycoprotein in cancer.

## Abbreviations

ADCC: antibody-dependent cellular cytotoxicity
ADCP: antibody-dependent cellular phagocytosis
AML: acute myeloblastic/myelogenous leukaemia
AuDC: autologous dendritic cell
CARs: chimeric antigen receptors
*MDSC*: *myeloid-derived suppressor cells*
DCs: dendritic cells
EC: extracellular domain
EGFR: epidermal growth factor receptor
KLH: keyhole limpet hemocyanin
MST: mean survival time
MUC1: mucin 1
NK: natural killer
NSCLC: non-small cell lung cancer
TR: tandem repeat
WT: wild type
